# Supplementary material for: Anxious individuals shift emotion control from lateral frontal pole to dorsolateral prefrontal cortex
Source: Nat Commun. 2023 Aug 12;14:4880. doi: 10.1038/s41467-023-40666-3 (PMC10423291; doi:10.1038/s41467-023-40666-3)
Supplement: Supplementary file 1 — Supplementary information [file 41467_2023_40666_MOESM1_ESM.pdf]

Supplementary information for:

Anxious individuals shift emotion control from lateral frontal pole to dorsolateral prefrontal cortex

Bob Bramson[1,2,T], Sjoerd Meijer [1,T], Annelies van Nuland[1], Ivan Toni[1,*] and Karin Roelofs[1,2,*]

Supplementary results

Extended congruency effects

In addition to the effects on error rates reported in the main manuscript, participants were slower in incongruent (M = 658 ms, std = 137) as compared to the congruent trials (M = 616 ms, std = 116), *b = 40 (ms) CI = [30 50]* (Supplementary figure 1). These effects confirm previous behavioral findings [1–3] and illustrate the validity of this task in evoking control costs when participants need to override automatic emotional action tendencies in favor of alternative actions. There were no interactions between congruency and group (non-anxious versus anxious) for reaction times, and no interactions with GABA/Glx ratios or amygdalofugal connectivity.

There were between groups differences in congruency effects (whole brain corrected) in SMC and fusiform cortex, Supplementary figure 2B, where congruent condition was stronger than incongruent in non-anxious as compared to the high-anxious group. All other significant clusters are described in table 1.

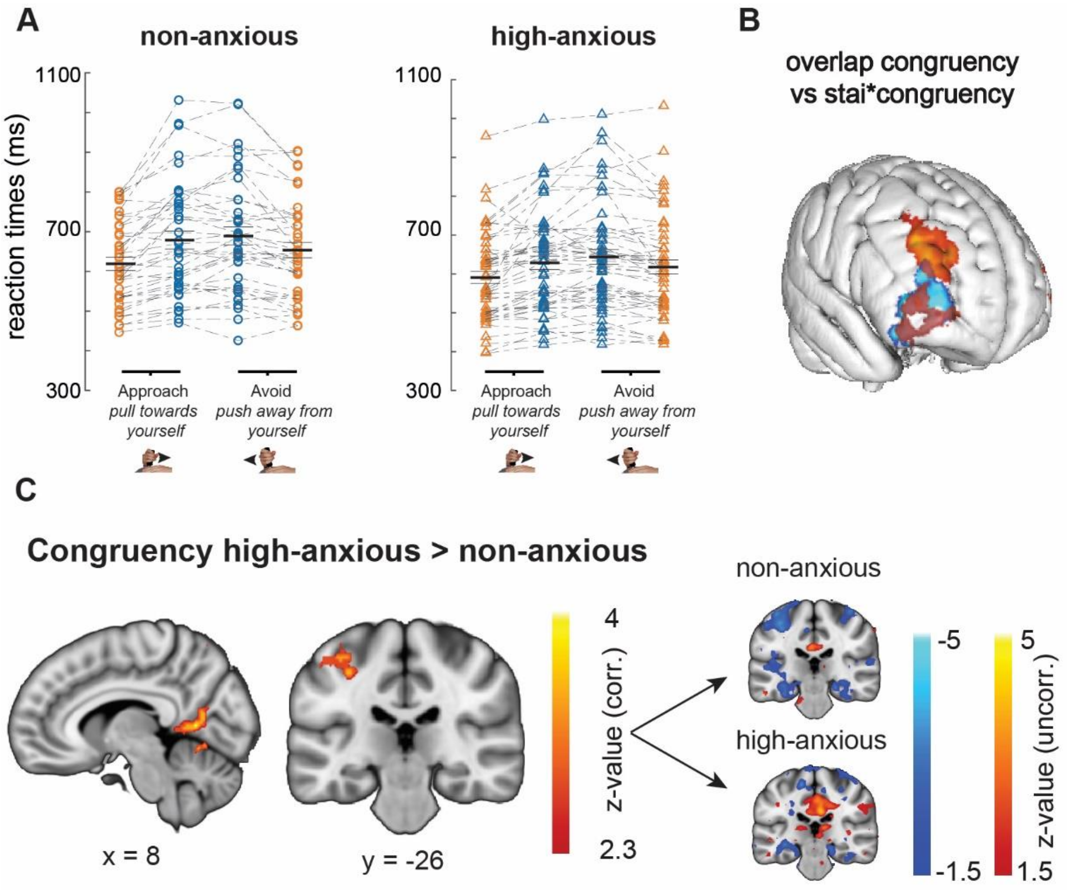

Supplementary figure 1. Reaction time and neural congruency effects between groups.

A) Reaction time congruency effects were not different between high-anxious and non-anxious groups. B) Overlap of neural congruency effect (hot colormap) and STAI*neural congruency (in blue. Overlapping voxels are plotted in purple. C)there were whole brain corrected differences between groups in neural congruency effects in SMC and Fusiform cortex. Right panels show uncorrected

brain figures to interpret those effects. Non-anxious group shows stronger activity in congruent versus incongruent conditions (in blue) in SMC that is not present in the high-anxious group.

Extended GABA/Glx analyses

To be able to draw inferences about the anatomical selectivity of the relationship between behavioral congruency effects GABA/Glx ratios in FPl and SMC, we estimated GABA/Glx ratios from occipital cortex (supplementary figure 3). GABA/Glx ratios were not different between groups for SMC and occipital cortex, suggesting that the increased excitability for high-anxiety participants is anatomically specific to FPl, supplementary figure 2A. In contrast to GABA/Glx ratios extracted from FPl and SMC (main figure 3; supplementary figure 2B), in occipital cortex congruency effects in error rates did not depend on GABA/Glx ratios derived from occipital cortex, *b = .02 CI [-.07 .11].* This finding indicates that the relationship between GABA/Glx ratios and congruency effects is anatomically specific.

Teasing apart the differential effect of FPl GABA/Glx ratio on neural congruency effects between groups (figure 3; supplementary figure 2C;D.) showed that, in the non-anxious group, neural congruency effects in SMC and parietal cortices were negatively correlated to FPl GABA/Glx ratios. In contrast, those effects were absent in the high-anxious group, supplementary figure 2D. Furthermore, in the non-anxious group, participants with lower GABA/glx ratio showed comparatively stronger activity in the congruent than in the incongruent condition. This suggests that the ability to recruit FPl determines the influence this region can have on SMC activation [1,4]. The high-anxious group did not show this correlation, which mirrors the behavioral effects.

Correlating SMC GABA/Glx ratio with BOLD congruency effects showed differential relationships between non-anxious and high-anxious participants in left SMC [-24 -24 68] and right dorsal premotor cortex [16 4 50]. This was mainly attributable to a significant correlation between congruency in SMC in the non-anxious group, where more excitable SMC was related to reduced congruency effects again mirroring the correlations between GABA/Glx ratio and behavioral congruency.

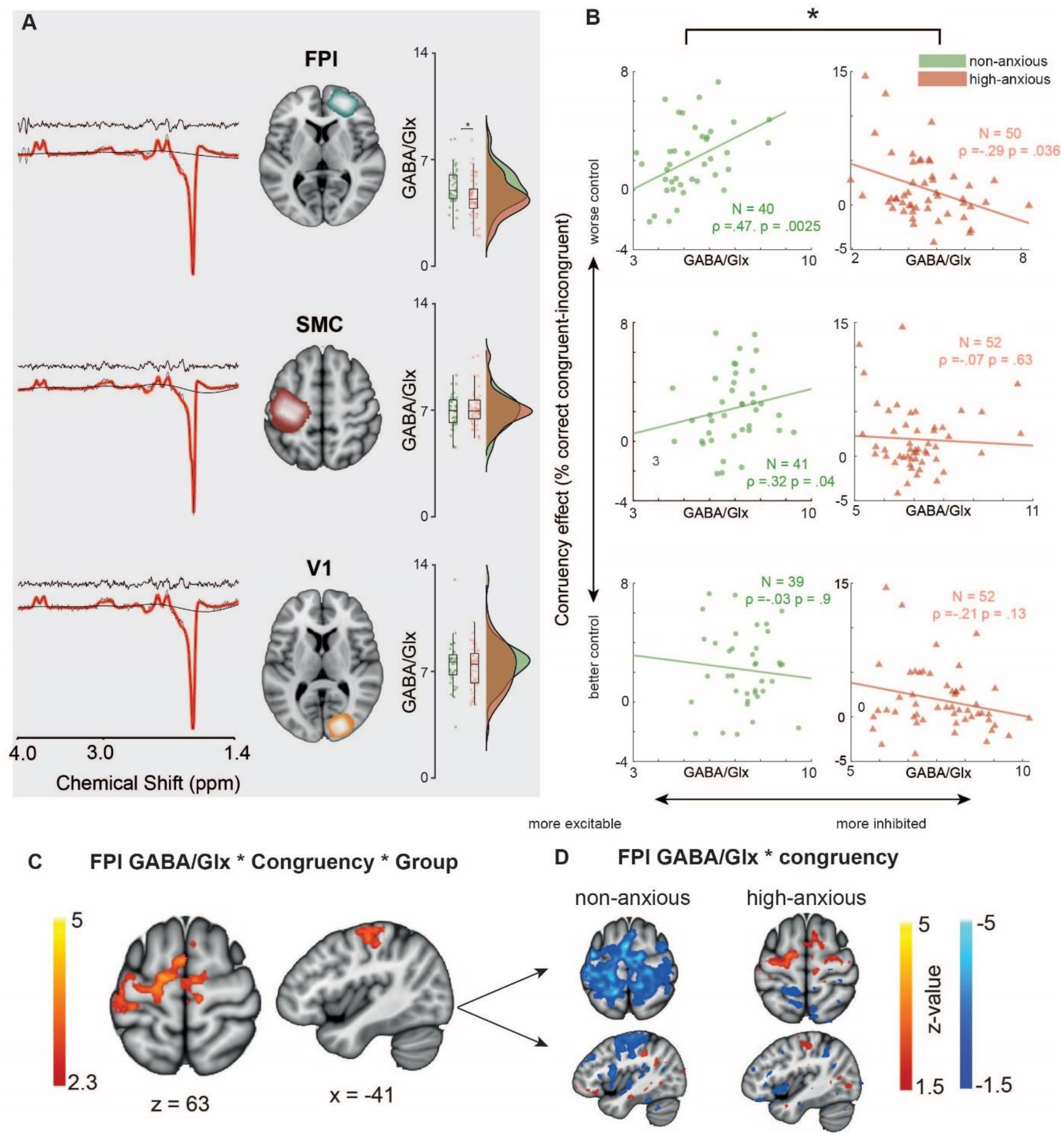

Supplementary figure 2. Correlations between GABA/Glx ratios and behavioral and neural congruency effects.

A) MRS example spectra (left) and average placement (right) for all three regions tested. GABA/Glx ratios were lower (more excitation) for high-anxious (N = 50) as compared to non-anxious in FPl (N = 40), but not in SMC (N = 52 high-anxious and N = 41 non-anxious) and occipital cortex (N = 52 high-anxious and N = 39 non-anxious). B) GABA/Glx ratios in FPl correlated differently with behavioral congruency for non-anxious versus high-anxious groups but not for SMC and occipital cortex. Figures show uncorrected p-values, after Bonferroni correction for multiple comparisons across groups only the correlation between FPl GABA/Glx ratio and behavioral congruency remains significant, p = .0075. C) GABA/Glx ratios from FPl correlated differently with neural congruency effects in left SMC for non-anxious as compared to high-anxious participants, mirroring the behavioral effects. D) non-thresholded plots show that this effect is attributable to strong negative correlations between congruency effects and FPl GABA/Glx ratio in non-anxious, but not in anxious participants.

To assess which regressors might explain shared variance in the behavioral congruency we performed a Bayesian mixed effects model explaining performance based on a six-way interaction between *Congruency (congruent/incongruent) * Group (non/high-anxious)*FPl engagement (BOLD effect congruentVSIncongruent) * dPFC engagement * FPl GABA/Glx * amygdalofugal-FPl connectivity*. This model results in a significant *Congruency*Group*FPl GABA/Glx* interaction; *b = .2, CI [.04 .37]*. However, several interactions we observed earlier are no longer statistically reliable. Given that *Congruency*Group*amygdalofugal connectivity* (figure 4B), and *Congruency*Group*dPFC BOLD* were significant when considered in isolation, we infer that *dPFC congruency* and *amygdalofugal-FPl connectivity* explain shared variance in the congruency effects on behavior. Accordingly, amygdalofugal-FPl projections correlate to dPFC engagement (figure 4D).

However, while *amygdalofugal-FPl connectivity* and *dPFC BOLD congruency* explain shared variance, this shared variance is at least partly separate from the variance explained by *FPl GABA/Glx*. This is intuitive given that GABA/Glx ratio in FPl explains more variance in behavior in non-anxious (figure 3B), whereas high-anxious recruit dPFC rather than FPl. The extent of this compensatory recruitment depends on the strength of the amygdalofugal projection strength (Figure 4D).

Extended diffusion analyses

In contrast to connections between amygdalofugal pathway and FPl we did not observe group differences between high-anxious and non-anxious individuals for area FPm; $t(90) = 1.08$, $p=.28$, area 24: $t(90) = -.007$, $p=.99$ and area 25: $t(90) = -.75$, $p=.45$.

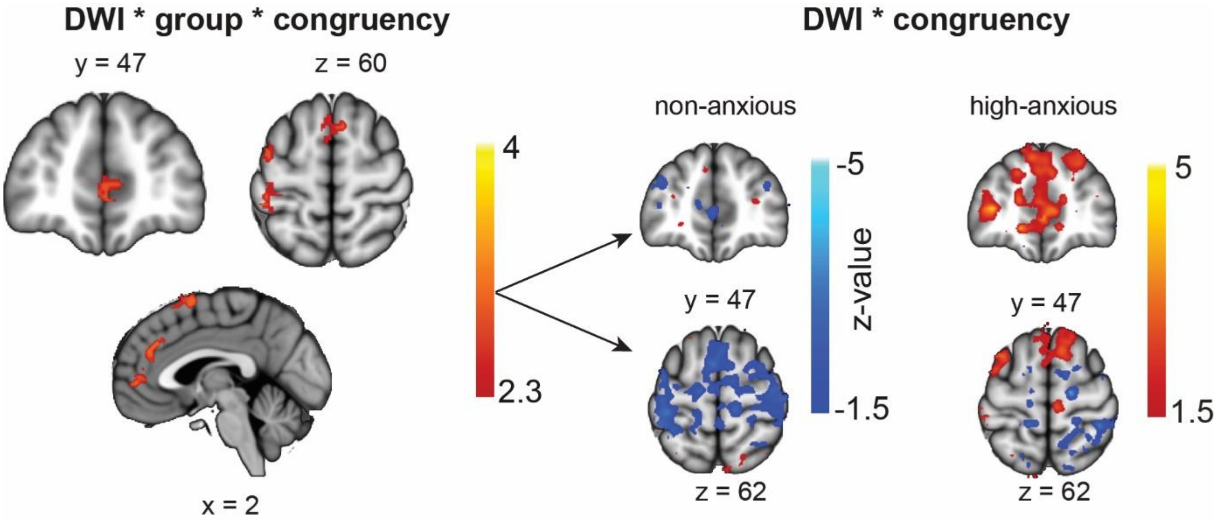

Supplementary figure 3. Anatomically specific amygdalofugal connections to FPl correlate with anxiety scores and neural indices of emotion control.

*There was an interaction between congruency*group*amygdalofugal tract strength. This indicates that there are differences in the way amygdalofugal projections to FPl correlated with neural congruency effects between non-anxious and high-anxious groups. These differences stem from stronger correlations between amygdalofugal tract strength and neural congruency in medial prefrontal cortex for the high-anxiety group (rightmost panel) as compared to the non-anxious group.*

Supplementary table 1: Neural congruency effects for different contrasts.

**Congruency both groups**				
Contrast: Congruent<Incongruent				
Region	max X	max Y	max Z	max z-value
Precuneus (L)	-12	-72	40	5.59
Frontal Pole (R)	38	42	30	4.7
Middle Frontal Gyrus (L)	-40	34	30	4.54
Cingulate Gyrus (L)	-6	-34	24	5.33
Contrast: Incongruent<Congruent				
	max X	max Y	max Z	max z-value
Frontal Pole	0	56	-8	5.04
Hippocampus (L)	-20	-20	-20	4.64
Fusiform Cortex (R)	40	-44	-20	4.31
Postcentral Gyrus (L)	-32	-30	70	3.5
Postcentral Gyrus (R)	42	-26	68	4.33
Precuneus (L)	-2	-58	16	3.9
Lateral Occipital cortex (L)	-44	-78	-4	3.67

**non-anxious**				
Contrast: Congruent<Incongruent				
Region	max X	max Y	max Z	max z-value
Supramarginal Gyrus (L)	-64	-46	38	4.92
Middle Frontal Gyrus (L)	-42	34	28	4.25
Angular Gyrus (R)	64	-48	42	3.74
Frontal Pole (R)	40	40	28	4.58
Cingulate Gyrus (L)	-6	-34	24	4.02
Precuneus (R)	8	-76	44	3.99
Precuneus (L)	-8	-74	40	4.79
Contrast: Incongruent<Congruent				
	max X	max Y	max Z	max z-value
Lateral Occipital cortex (R)	54	-74	-8	4.92
Lingual Gyrus (L)	-26	-42	-12	4.57
Precentral Gyrus (L)	-38	-14	54	4.18
Frontal Medial Cortex (R)	4	52	-8	4.87
Temporal Pole (R)	44	20	-30	4.41
Postcentral Gyrus (R)	40	-24	68	4.14
Occipital Pole (L)	-28	-94	-18	4.04
Planum Temporale (R)	64	-10	6	3.93

**high-anxious**				
Contrast: Congruent<Incongruent				
Region	max X	max Y	max Z	max z-value
Precuneus (R)	14	-70	36	4.98
Middle Frontal Gyrus (R)	32	34	34	4.72
Paracingulate Gyrus (R)	8	32	28	3.82
Contrast: Incongruent<Congruent				
	max X	max Y	max Z	max z-value
Temporal Pole (L)	-42	16	-36	4.68
Frontal Orbital cortex (R)	24	30	-14	4.2
Frontal Medial Cortex (L)	-8	34	-14	4.02
Superior Temporal Gyrus (R)	38	-4	-20	3.88
Fusiform Cortex (R)	40	-44	-20	4.31
Frontal Orbital cortex (L)	-40	28	-14	3.83

**Congruency * group**				
Contrast: high-anxious(conVSinc) > non-anxious(conVSinc)				
Region	max X	max Y	max Z	max z-valu
Lingual Gyrus (R)	22	-50	-12	3.86
Superior Parietal Lobule (R)	26	-46	58	3.74
Precentral Gyrus (L)	-32	-16	68	3.34

Supplementary table 2: Correlations between GABA/Glx ratios from FPl and SMC and neural congruency effects.

both groups				
*FPl GABA/Glx * Congruency*				
Region	*max X*	*max Y*	*max Z*	*max z-value*
Postcentral Gyrus (L)	-14	-44	64	4.27

non-anxious				
*FPl GABA/Glx * congruency (negative)*				
Region	*max X*	*max Y*	*max Z*	*max z-value*
Postcentral Gyrus (R)	18	-28	70	4.57
Precentral Gyrus (R)	60	8	30	4.67
Precentral Gyrus (L)	-54	-2	20	3.93
*SMC GABA/Glx*congruency*				
	max X	*max Y*	*max Z*	*max z-value*
Precentral Gyrus (R)	16	-24	72	3.98

high-anxious				
*SMC GABA/Glx * congruency (negative)*				
Region	*max X*	*max Y*	*max Z*	*max z-value*
Frontal Pole (R)	54	36	16	3.49
Occipital Pole (R)	24	-92	34	3.68

Congruency * group				
*FPl GABA/Glx * Congruency * group*				
Region	*max X*	*max Y*	*max Z*	*max z-valu*
Supplementary Motor Area (L)	-6	0	54	4.18
*SMC GABA/Glx * Congruency * group*				
	max X	*max Y*	*max Z*	*max z-valu*
Precentral Gyrus (L)	-10	-26	66	3.85
Supplementary Motor Area (R)	16	4	50	4.24

Supplementary table 3: Correlations between amygdalofugal tract strength and neural congruency effects.

both groups				
*Amygdalofugal*congruency (negative)*				
Region	*max X*	*max Y*	*max Z*	*max z-value*
Postcentral Gyrus (R)	42	-16	28	3.84
Postcentral Gyrus (R)	20	-38	70	3.69

non-anxious				
*amygdalofugal*congruency(negative)*				
Region	*max X*	*max Y*	*max Z*	*max z-value*
Precentral Gyrus (L)	-10	-12	72	4.12
Postcentral Gyrus (R)	48	-14	58	4.1
Precentral Gyrus (L)	-50	4	36	4.45

high-anxious				
*amygdalofugal*congruency*				
	max X	*max Y*	*max Z*	*max z-value*
Medial Frontal Cortex (L)	-14	46	-6	4.1
Occipital Pole (R)	16	-88	20	3.73
Middle Temporal Gyrus (L)	-68	-14	-10	3.75

Congruency * group				
*Amygdalofugal*group*congruency*				
Region	*max X*	*max Y*	*max Z*	*max z-valu*
Superior Frontal Gyrus (L)	-4	8	70	3.65
Paracingulate Gyrus	0	50	2	3.59
Precentral Gyrus (L)	-42	0	54	3.62
Temporal Pole (L)	-62	4	-16	3.46

Main statistical models used on correct responses	*b*	*CI*		*significant'*
Group * Congruency	0.034	-0.055	0.12	no
Group	0.12	-0.04	0.28	no
Congruency	0.2	0.1	0.29	yes
Group * Congruency * FPl GABA/Glx * SMC GABA/Glx	-0.1	-0.21	-0.0002	yes
Group * Congruency * FPl GABA/Glx	0.19	0.1	0.29	yes
Group * Congruency * SMC GABA/Glx	0.04	-0.04	0.14	no
Group * Congruency * amygdalofugal projections	0.14	0.02	0.26	yes
Models split out between groups				
High-anxious				
Congruency* FPl GABA/Glx * SMC GABA/Glx	0.07	-0.04	0.19	no
Congruency* FPl GABA/Glx	-0.13	-0.25	-0.006	yes
Congruency * SMC GABA/Glx	-0.02	-0.13	0.1	no

Non-anxious				
Congruency* FPl GABA/Glx * SMC GABA/Glx	0.13	-0.29	0.04	no
Congruency*FPl GABA/Glx	0.24	0.12	0.38	yes
Congruency * SMC GABA/Glx	0.05	-0.08	0.19	no

Supplementary table 4; statistical models run and most important interactions.

This table highlights the models that were run on the behavioral data and the most important interactions that resulted from those models.

References

1. Bramson, B., Jensen, O., Toni, I. & Roelofs, K. Cortical oscillatory mechanisms supporting the control of human social-emotional actions. *J. Neurosci.* 3317–3382 (2018).
2. Volman, I., Roelofs, K., Koch, S., Verhagen, L. & Toni, I. Anterior prefrontal cortex inhibition impairs control over social emotional actions. *Curr. Biol.* **21**, 1766–1770 (2011).
3. Kaldewaij, R. *et al.* Anterior prefrontal brain activity during emotion control predicts resilience to post-traumatic stress symptoms. *Nat. Hum. Behav.* **5**, 1055–1064 (2021).
4. Voytek, B. *et al.* Oscillatory dynamics coordinating human frontal networks in support of goal maintenance. *Nat. Neurosci.* (2015).

List of images used in the AAT, taken from KDEF & AKDEF (https://kdef.se/)

AM01HAS.bmp

AM02HAS.bmp

AM06HAS.bmp

AM08HAS.bmp

AM09HAS.bmp

AM10HAS.bmp

AM11HAS.bmp

AM21HAS.bmp

AM22HAS.bmp

AM03HAS.bmp

AM29HAS.bmp

AM34HAS.bmp

AM14HAS.bmp

EFS022.bmp

EFS029.bmp

EFS035.bmp

EFS074.bmp

EFS101.bmp

AF01HAS.bmp

AF02HAS.bmp

AF03HAS.bmp

AF05HAS.bmp

AF14HAS.bmp

AF18HAS.bmp

AF20HAS.bmp

AF21HAS.bmp

AF26HAS.bmp

AF29HAS.bmp

AF35HAS.bmp

EFS001.bmp

EFS007.bmp

EFS042.bmp

EFS048.bmp

EFS057.bmp

EFS066.bmp

FS085.bmp

AM01ANS.bmp

AM02ANS.bmp

AM06ANS.bmp

AM08ANS.bmp

AM09ANS.bmp

AM10ANS.bmp

AM11ANS.bmp

AM21ANS.bmp

AM22ANS.bmp

AM03ANS.bmp

AM29ANS.bmp

AM34ANS.bmp

AM14ANS.bmp

EFS025.bmp

EFS030.bmp

EFS038.bmp

EFS080.bmp

EFS105.bmp

AF01ANS.bmp

AF02ANS.bmp

AF03ANS.bmp

AF05ANS.bmp

AF14ANS.bmp

AF18ANS.bmp

AF20ANS.bmp

AF21ANS.bmp

AF26ANS.bmp

AF29ANS.bmp

AF35ANS.bmp

EFS003.bmp

EFS010.bmp

EFS044.bmp

EFS052.bmp

EFS062.bmp

EFS069.bmp

EFS089.bmp
