# Supplementary material for: Anxious individuals shift emotion control from lateral frontal pole to dorsolateral prefrontal cortex
Source: Nat Commun. 2023 Aug 12;14:4880. doi: 10.1038/s41467-023-40666-3 (PMC10423291; doi:10.1038/s41467-023-40666-3)
Supplement: Supplementary file 3 — Reporting Summary [file 41467_2023_40666_MOESM3_ESM.pdf]

nature portfolio

Corresponding author(s): Bob Bramson

Last updated by author(s): Jun 9, 2023

Reporting Summary

Nature Portfolio wishes to improve the reproducibility of the work that we publish. This form provides structure for consistency and transparency in reporting. For further information on Nature Portfolio policies, see our Editorial Policies and the Editorial Policy Checklist.

Statistics

For all statistical analyses, confirm that the following items are present in the figure legend, table legend, main text, or Methods section.

n/a	Confirmed	
☐	☒	The exact sample size (*n*) for each experimental group/condition, given as a discrete number and unit of measurement
☐	☒	A statement on whether measurements were taken from distinct samples or whether the same sample was measured repeatedly
☐	☒	The statistical test(s) used AND whether they are one- or two-sided<br>*Only common tests should be described solely by name; describe more complex techniques in the Methods section.*
☐	☒	A description of all covariates tested
☐	☒	A description of any assumptions or corrections, such as tests of normality and adjustment for multiple comparisons
☐	☒	A full description of the statistical parameters including central tendency (e.g. means) or other basic estimates (e.g. regression coefficient) AND variation (e.g. standard deviation) or associated estimates of uncertainty (e.g. confidence intervals)
☐	☒	For null hypothesis testing, the test statistic (e.g. *F*, *t*, *r*) with confidence intervals, effect sizes, degrees of freedom and *P* value noted<br>*Give P values as exact values whenever suitable.*
☐	☒	For Bayesian analysis, information on the choice of priors and Markov chain Monte Carlo settings
☐	☒	For hierarchical and complex designs, identification of the appropriate level for tests and full reporting of outcomes
☐	☒	Estimates of effect sizes (e.g. Cohen's *d*, Pearson's *r*), indicating how they were calculated

Our web collection on statistics for biologists contains articles on many of the points above.

Software and code

Policy information about availability of computer code

Data collection	Presentation Neurobs Software; version 16.4. https://www.neurobs.com/
Data analysis	matlab2020b https://www.mathworks.com/<br>R version 4.2.2 https://www.r-project.org/<br>JASP https://jasp-stats.org/<br>LCmodel http://s-provencher.com/lcmodel.shtml<br>FSL 6.0.0 https://fsl.fmrib.ox.ac.uk/fsl/fslwiki<br>EDDY https://fsl.fmrib.ox.ac.uk/fsl/fslwiki/eddy<br>BedpostX https://fsl.fmrib.ox.ac.uk/fsl/fslwiki/FDT/UserGuide#BEDPOSTX

For manuscripts utilizing custom algorithms or software that are central to the research but not yet described in published literature, software must be made available to editors and reviewers. We strongly encourage code deposition in a community repository (e.g. GitHub). See the Nature Portfolio guidelines for submitting code & software for further information.

Data

Policy information about availability of data

All manuscripts must include a data availability statement. This statement should provide the following information, where applicable:

- Accession codes, unique identifiers, or web links for publicly available datasets
- A description of any restrictions on data availability
- For clinical datasets or third party data, please ensure that the statement adheres to our policy

All data reported in this manuscript can be downloaded from the donders data repository https://data.donders.ru.nl/

Research involving human participants, their data, or biological material

Policy information about studies with human participants or human data. See also policy information about sex, gender (identity/presentation), and sexual orientation and race, ethnicity and racism.

Reporting on sex and gender	Fifty-two high-anxious (13 males) and forty-four non-anxious (all males) students of the Radboud University Nijmegen participated in this experiment after giving informed consent.
Reporting on race, ethnicity, or other socially relevant groupings	We did not ask for race, ethnicity or other groupings except for anxiety questionnaires.
Population characteristics	Fifty-two high-anxious (13 males) and forty-four non-anxious (all males) students of the Radboud University Nijmegen participated in this experiment after giving informed consent. Two non-anxious participants were excluded because they did not attend the whole experiment; one participant was excluded because they failed to comply with the task instructions. All participants had normal or corrected to normal vision and were screened for contra-indications for magnetic resonance imaging. Participants mean age for non-anxious: 23.8 years, SD = 3.4, range 18-34; for high-anxious mean = 25.66, SD = 4.4, range 20-39. The analyses and sample size for the high-anxious were preregistered at:https://osf.io/j9s2z/?view_only=5510570459694d619adb5dca4019e9fa, announcing two analyses: a brain-stimulation analysis that is not part of the current paper and the planned comparison between the high-anxious and non-anxious group on amygdalofugal projections to FPl and GABA/Glx interactions with behavior reported here). Data from the high-anxious sample have not been reported on previously.
Recruitment	Participants were recruited by signing up via the University recruitment website. This means that mainly university students were recruited, potentially limiting the generalization of results.
Ethics oversight	The study was approved by the local ethics committee (CMO:2014/288).

Note that full information on the approval of the study protocol must also be provided in the manuscript.

Field-specific reporting

Please select the one below that is the best fit for your research. If you are not sure, read the appropriate sections before making your selection.

[x] Life sciences [ ] Behavioural & social sciences [ ] Ecological, evolutionary & environmental sciences

For a reference copy of the document with all sections, see nature.com/documents/nr-reporting-summary-flat.pdf

Life sciences study design

All studies must disclose on these points even when the disclosure is negative.

Sample size	Fifty-two high-anxious (13 males) and forty-four non-anxious (all males) students of the Radboud University Nijmegen participated in this experiment after giving informed consent. Sample size rationale can be found at https://osf.io/j9s2z/wiki/home/?view_only=5510570459694d619adb5dca4019e9fa and https://osf.io/m9bv7/wiki/home/?view_only=18d58e2351b14584b6e688599472534e
Data exclusions	Two non-anxious participants were excluded because they did not attend the whole experiment; one participant was excluded because they failed to comply with the task instructions.
Replication	FPl involvement in emotional-action control has been replicated often, e.g. Bramson et al., 2020 - Jneuro<br>Replication of MRS findings was not possible due to lack of available data, time & money.
Randomization	trials and block order were randomized across participants. randomization across groups was not possible given the aim of the study.
Blinding	participants were blind to the goal of the study

Reporting for specific materials, systems and methods

We require information from authors about some types of materials, experimental systems and methods used in many studies. Here, indicate whether each material, system or method listed is relevant to your study. If you are not sure if a list item applies to your research, read the appropriate section before selecting a response.

Materials & experimental systems

n/a	Involved in the study
☒	☐ Antibodies
☒	☐ Eukaryotic cell lines
☒	☐ Palaeontology and archaeology
☒	☐ Animals and other organisms
☒	☐ Clinical data
☒	☐ Dual use research of concern
☒	☐ Plants

Methods

n/a	Involved in the study
☒	☐ ChIP-seq
☒	☐ Flow cytometry
☐	☒ MRI-based neuroimaging

Magnetic resonance imaging

Experimental design

Design type

Task; Event related; Structural/trait measures

Design specifications

Written instructions were presented on the screen for a minimum of 30 seconds prior to the start of each block of 12 trials. Each participant performed 288 trials on each of the two testing days, yielding 576 trials in total, equally divided between congruent and incongruent conditions.

Behavioral performance measures

For all behavior analyses we focused primarily on differential error rates between the congruent and incongruent condition, because those have been most strongly linked to both individual differences in structural and functional properties of the FPl system under investigation. Reaction time analyses are reported in the supplementary materials.

Acquisition

Imaging type(s)

functional; structural; diffusion; spectroscopy

Field strength

3

Sequence & imaging parameters

All magnetic resonance images were acquired using a 3T MAGNETROM Prisma MR scanner (Siemens AG, Healthcare Sector, Erlangen, Germany) using a 32-channel headcoil for the structural T1 and MRS scans, and a 64-channel headcoil for the functional images.

High-resolution anatomical images were acquired with a single-shot MPRAGE sequence with an acceleration factor of 2 (GRAPPA method), a TR of 2400 ms, TE 2.13 ms. Effective voxel size was 1 x 1 x 1 mm with 176 sagittal slices, distance factor 50%, flip angle 8°, orientation A ≫ P, FoV 256 mm.

Magnetic resonance images were acquired using a MEGA-PRESS WIP sequence (SIEMENS) with TE = 68 ms, TR = 1500 ms, water suppression at 4.7 ppm (CHESS81 and acquisition bandwidth of 1200 Hz. In one of every two acquisitions, a refocusing pulse was applied at 1.9 ppm. Subtracting these signals from the non-refocused scans showed GABA resonance at 3.00 ppm. As a proxy for Glutamate levels we used Glx, which consists of combined Glutamate and Glutamine levels. Glx was estimated from unedited spectra following earlier protocols82. MRS measurements were acquired from right FPl 20,27, and from left SMC 3. A voxel in the right occipital cortex was also measured as a non-task-related control. Voxel size was 1.8 x 1.8 x 2.5 cm82.

The field of view of the functional scans acquired in the MR-sessions was aligned to a built-in brain-atlas to ensure a consistent MR field of view across days. Approximately 1800 functional images were continuously acquired in each scanning day using a multi-band 6 sequence, 2*2*2 mm voxel size, TR/TE=1000/34ms, Flip angle = 60º, phase angle P>>A, including 10 volumes with reversed phase encoding (A>>P) to correct image distortions.

Area of acquisition

whole brain; MRS voxels placed at right prefrontal cortex; left sensorimotor cortex and left visual cortex

Diffusion MRI ☒ Used ☐ Not used

Parameters

Diffusion-weighted images were acquired using echo-planar imaging with multiband acceleration factor of 2 (GRAPPA method), multiband acceleration factor = 3. We acquired 93 1.6 mm thick transversal slices with voxel size of 1.6 x 1.6 x 1.6 mm, phase encoding direction A >> P, FoV 211 mm, TR = 3350, TE = 71.20. 256 isotropically distributed directions were acquired using a b-value of 2500 s/mm2. An additional volume without diffusion weighting with reverse phase encoding (P >> A) was also acquired.

Preprocessing

Preprocessing software

fMRI images were analysed using FSL 6.0.0 (https://fsl.fmrib.ox.ac.uk). Images were motion corrected using MCFLIRT87, and distortions in the magnetic field were corrected using TOPUP88. Functional images were rigid-body registered to the brain extracted structural image using FLIRT. Registration to MNI 2 mm standard space used the nonlinear registration tool FNIRT. Images were spatially smoothed using a Gaussian 5 mm kernel and high pass filtered with a cut-off estimated on the task

structure. Independent component analysis was run with a pre-specified maximum of 100 components89; these components were manually inspected to remove potential sources of noise.

Diffusion data was preprocessed using FSL FDT 3.0 (https://fsl.fmrib.ox.ac.uk). Susceptibility artefacts were estimated using TOPUP using additional b = 0 volumes with reverse phase coding direction88. Data were then corrected for potential distortions during eddy currents and movement by using the EDDY tool92. Crossing fibers were estimated using BedpostX with default settings93.

Spectroscopy data were analyzed using LCmodel software83. After frequency alignment, eddy current correction, phase- and baseline corrections, the relative concentrations of neurotransmitters were estimated using basis sets, against which we fitted the acquired signals in both the edited and non-edited spectra. Spectra quality control was based on several estimates of signal quality; the % SD provided by LCmodel, which reflects the Cramer-Rao lower bound; full-width half maximum FWHM, estimates of signal to noise ratio provided by LCmodel and visual inspection. Inhibitory tone was then calculated by calculating the ratio between GABA and GLx.

Normalization

Functional images were rigid-body registered to the brain extracted structural image using FLIRT. Registration to MNI 2 mm standard space used the nonlinear registration tool FNIRT.

Normalization template

MNI152 standard-space T1-weighted average

Noise and artifact removal

). Images were motion corrected using MCFLIRT87, and distortions in the magnetic field were corrected using TOPUP88. Functional images were rigid-body registered to the brain extracted structural image using FLIRT. Registration to MNI 2 mm standard space used the nonlinear registration tool FNIRT. Images were spatially smoothed using a Gaussian 5 mm kernel and high pass filtered with a cut-off estimated on the task structure. Independent component analysis was run with a pre-specified maximum of 100 components89; these components were manually inspected to remove potential sources of noise.

Volume censoring

N/A

Statistical modeling & inference

Model type and settings

First and second level GLM analyses were performed using FEAT 6.00 implemented in FSL 6.0.0. The first-level model consisted of twelve task regressors: Approach angry, approach happy, avoid angry and avoid happy trials were modelled separately for each of the three stimulation conditions (for details on stimulation conditions see Bramson et al., 2020a). In each regressor, each event covered the time interval from presentation of a face until the corresponding onset of the joystick movement. Estimated head translations/rotations during scanning (six regressors), temporal derivatives of those translations/rotations (six regressors), and MR-signals in white matter and cerebrospinal fluid (2 regressors) were included to the GLM as nuisance covariates. Emotional control effects were estimated by comparing incongruent trials (approach angry and avoid happy) to congruent trials. First level models of the two separate sessions were combined using Fixed Effects analyses implemented in FEAT.
Whole brain group effects and their relationship to GABA/Glx ratio's in FPl and SMC, and amygdalofugal connections to FPl were assessed using FLAME 1 with outlier de-weighting90, making family-wise error corrected cluster-level inferences using a cluster-forming threshold of z > 2.3. This threshold provides a false error rate of around 5% when using FSL's FLAME 191. In both whole brain and ROI analyses we used standardized GABA/Glx ratio's extracted from FPl and SMC as regressors. GABA/Glx ratio extracted from occipital cortex was used in a separate control analysis.

Effect(s) tested

Congruency
Congruency*Group
Congruency*Group*DTI
Congruency*Group*FPl GABA/Glx
Congruency*Group*trait anxiety score

Specify type of analysis: ☐ Whole brain ☐ ROI-based ☒ Both

Anatomical location(s) Frontal lobe (Harvard-Oxford atlas provided by FSL)

Statistic type for inference

(See Eklund et al. 2016)

Whole brain group effects and their relationship to GABA/Glx ratio's in FPl and SMC, and amygdalofugal connections to FPl were assessed using FLAME 1 with outlier de-weighting90, making family-wise error corrected cluster-level inferences using a cluster-forming threshold of z > 2.3. This threshold provides a false error rate of around 5% when using FSL's FLAME 191.

Correction

Whole brain group effects and their relationship to GABA/Glx ratio's in FPl and SMC, and amygdalofugal connections to FPl were assessed using FLAME 1 with outlier de-weighting90, making family-wise error corrected cluster-level inferences using a cluster-forming threshold of z > 2.3. This threshold provides a false error rate of around 5% when using FSL's FLAME 191.

Models & analysis

n/a	Involved in the study
☒	☐ Functional and/or effective connectivity
☒	☐ Graph analysis
☒	☐ Multivariate modeling or predictive analysis
